# Imaging stretch-activated firing of spinal afferent nerve endings in mouse colon

**DOI:** 10.3389/fnins.2013.00179

**Published:** 2013-10-07

**Authors:** Lee Travis, Nick J. Spencer

**Affiliations:** Discipline of Human Physiology, Center for Neuroscience, School of Medicine, Flinders UniversityBedford Park, SA, Australia

**Keywords:** nerve terminal, afferent, colon

## Abstract

Spinal afferent neurons play a major role in detecting noxious and innocuous stimuli from visceral organs, such as the gastrointestinal tract. However, all our understanding about spinal afferents has been obtained from recordings of spinal afferent axons, or cell bodies that lie outside the gut wall, or peripheral organ they innervate. No recordings have been made directly from spinal afferent nerve endings, which is where sensory transduction occurs. We developed a preparation whereby recordings could be made from rectal afferent nerve endings in the colon, to characterize mechanisms underlying sensory transduction. Dorsal root ganglia (L6-S2) were removed from mice, whilst retaining neural continuity with the colon. Fluo-4-AM was used to record from rectal afferent nerve endings in myenteric ganglia and circular muscle at 36°C. In slack (unstretched) preparations of colon, no calcium transients were recorded from spinal afferent endings. However, in response to a maintained increase in circumferential diameter, a maintained discharge of calcium transients occurred simultaneously in multiple discrete varicosities along single axons of rectal afferents in myenteric ganglia and circular muscle. Stretch-activated calcium transients were resistant to hexamethonium and nifedipine, but were abolished by tetrodotoxin, CPA, BAPTA-AM, cobalt, gadolinium, or replacement of extracellular Na^+^ with NMDG. In summary, we present a novel preparation in which stretch-activated firing of spinal afferent nerve endings can be recorded, using calcium imaging. We show that circumferential stretch of the colon activates a maintained discharge of calcium transients simultaneously in varicosities along single rectal afferent endings in myenteric ganglia and circular muscle. Non-selective cation channels, TTX-sensitive Na^+^ channels and both extracellular calcium influx and intracellular Ca^2+^ stores are required for stretch-activated calcium transients in rectal afferent endings.

## Introduction

Remarkably few studies have recorded from sensory nerve terminals in vertebrates (Mendelson and Lowenstein, 1965; Firestein et al., 1990; Brock et al., 1998, 2001; Carr and Brock, 2002; Carr et al., 2009). In recent years, the cornea is perhaps the only region in the body in which recordings have been made directly from nociceptive nerve endings (Brock et al., 1998, 2001; Carr and Brock, 2002; Carr et al., 2009). In visceral organs, such as the gastrointestinal tract, no recordings have been made from spinal afferent nerve endings that encode noxious (painful) and innocuous stimuli. Instead, recordings from spinal afferents have been limited to axons or cell bodies which lie some distance away from the peripheral organ they innervate (Brierley et al., 2004; Spencer et al., 2008a; Song et al., 2009; Feng et al., 2010, 2012).

In the GI tract, extensive evidence has now been presented that noxious and innocuous stimuli from the GI-tract are detected and transmitted by spinal afferent neurons, whose cell bodies lie in dorsal root ganglia (DRG), but whose nerve endings lie within the wall of the GI-tract (Traub, 2000; Kyloh et al., 2011; Zagorodnyuk et al., 2011). Whilst much has been learnt from extracellular recordings of spinal afferents that lie outside the gut wall, this technique does not reveal direct information about the mechanisms underlying sensory transduction, since this process only occurs within the afferent nerve endings located within the peripheral organ. This we demonstrated by studies which showed that physiological stimuli applied to the sensory nerve endings of spinal afferents leads to the generation of action potentials (Zagorodnyuk et al., 2003; Spencer et al., 2008a; Song et al., 2009), but not when the same stimuli are applied to the axons of spinal afferents that lie away from the transduction sites (Zagorodnyuk et al., 2003; Spencer et al., 2008a). The other major difference between spinal afferent axonal recordings outside the gut wall and direct recordings from nerve endings, is that the expression of sensory ion channels, and receptors can be very different between the cell body of a sensory neuron; and its nerve endings that respond directly to stimuli (Ramirez and Pearson, 1990). In light of our limited understanding of the mechanisms underlying sensory transduction in spinal afferents, it was our intention to develop a preparation whereby recordings could be made directly from spinal afferent endings, to characterize mechanisms underlying their activation by circumferential stretch.

The origin of the pain pathway from the distal colon and rectum to the spinal cord has been the subject of considerable interest for many years. Recently, in live mice, we made sequential and selective lesions to each of the major extrinsic nerve pathways that transmit sensation between the large intestine and spinal cord (Kyloh et al., 2011). We showed that in response to noxious levels of colorectal distension, the evoked visceromotor response (VMR) was unaffected by lesions to the lumbar colonic or hypogastric nerves, but was abolished by lesions to the rectal nerves (Kyloh et al., 2011). This revealed that the rectal/pelvic afferent pathway, with DRG cell bodies in the lumbosacral pathway, was the primary pain pathway for the transduction and transmission of visceral pain evoked by noxious colorectal distension, consistent with the findings of (Traub, 2000). The significance of rectal afferents in the transduction of visceral pain from the colorectum was further highlighted in endothelin-3 (ET-3) mutant mice which failed to respond to noxious levels of colorectal distension. In these mice, it was found that there was a selective deficit in a specific class of low threshold, wide dynamic range rectal afferent, without any changes to other classes of afferents (such as high threshold afferents) (Spencer et al., 2008a,b; Zagorodnyuk et al., 2011). We have previously shown that circumferential stretch of the mouse colon potently activates low threshold, wide dynamic range rectal afferents, whose varicose nerve endings innervate myenteric ganglia and circular smooth muscle (Spencer et al., 2008a,b). This class of low threshold afferent has been shown to be activated by physiological levels of circumferential stretch and exogenous capsaicin and generates increased firing of action potentials into the noxious range (Spencer et al., 2008a,b).

Since the lumbosacral (Traub, 2000), rectal/pelvic afferent pathway (Kyloh et al., 2011; Zagorodnyuk et al., 2011) plays a major role in the transduction of noxious mechanical stimuli from the colorectum, we sought to develop an imaging technique that enabled us to record directly from the nerve endings of rectal afferents, which predominantly terminate in myenteric ganglia and circular muscle. In brief, we found that in response to maintained circumferential stretch of the colon, calcium transients discharged simultaneously in multiple varicosities along rectal afferent nerve endings. Opening of non-selective, stretch-activated ion channels, TTX-sensitive Na^+^ channels and both intracellular calcium stores and extracellular calcium influx are required for the discharge of calcium transients in primary afferent endings.

## Methods

### Preparation of tissues

C57BL/6 mice (20–90 days old) of either sex were euthanized humanely by inhalation of anaesthetic (Isoflurane 1 mL/L) followed by cervical dislocation. The use and treatment of animals was approved by the Institutional Animal Use and Care Committee at Flinders University of South Australia. The entire colon was removed from the pelvic cavity with all extrinsic nerves preserved to retain neural continuity with DRG between L6-S2. A midline incision was made along the mesenteric border and the longitudinal muscle sharp dissected away from half of the myenteric plexus, ensuring no damage to the spinal nerves entering the colorectum. The mucosa and submucosal plexus was then removed from the colon, in ice cold Krebs solution. The entire preparation was then pinned circular muscle down in a Sylgard-lined petri dish containing oxygenated Krebs-Ringer Buffer (KRB: see composition below) at room temperature.

### Confirmation that calcium induced fluorescence changes in the colon arise from spinal afferent nerve endings

**Figure 1:**
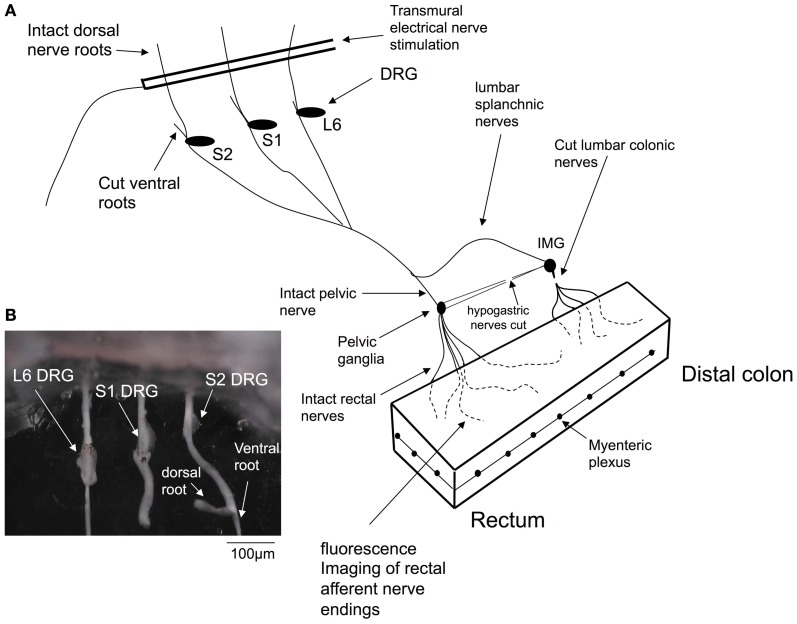
**Diagrammatic representation of the *in vitro* preparation used to image dynamic changes in intracellular calcium from spinal afferent nerve terminals in response to maintained circumferential stretch of the isolated mouse colon and rectum.** Panel **(A)**, shows a cartoon of the preparation used, where L6 to S2 DRGs were removed from either left or right side of the spinal cord and complete extrinsic neural continuity was retained with the large intestine. The mucosa and submucosal plexus was removed from the whole length of colon and part of the longitudinal muscle. Fine transmural electrical nerve stimulation wires were mounted over L6-S2 dorsal nerve roots to orthodromically activate spinal afferent endings, whilst imaging dynamic changes in cytosolic calcium in rectal nerve terminals. **(B)** Shows a photomicrograph of the DRGs (L6, S1 and S2) mounted in the organ bath chamber.

To confirm that stretch-activated calcium transients within the colon were generated in spinal afferent nerve endings, we created a preparation in which lumbosacral DRGs could be removed from the colon, whilst maintaining neural continuity with the colon. This is represented in Figure 1, where dorsal and ventral roots of L6-S2 DRG were isolated (see Figure 1A) and pinned to the base of the organ bath, whilst the whole colon was pinned (serosa side uppermost) to the base of a Sylgard-lined organ bath (8 mL) capacity. The organ bath was perfused with oxygenated Krebs solution at 36°C using a flow rate of ~2 mL/min. The mucosa and submucosal plexus was always removed from all colonic preparations. Fine transmural electrical stimulating wires (Teflon coated, platinum, 25 μm diameter) were mounted only over the dorsal roots (Figure 1). In some other experiments, we could also use a two chambered organ bath to separately perfuse agonists to the DRG chamber and examine the effects in the colon. In these types of experiments, we perfused a high K^+^ solution to the DRG chamber, to activated sensory nerve cell bodies, but not the endings directly. In both these types of preparations, we also made selective nerve lesions to the hypogastric and colonic nerves. This meant that any stimulation of the DRGs or dorsal roots could only activate rectal nerve terminals in the colon, and not nerve afferent terminals that arose from the thoracolumbar DRG, running in the lumbar splanchnic nerves (see Figure 1).

### Protocol for Ca^2+^ imaging from spinal afferent terminals

After the colon and DRG preparation was dissected and mounted in the organ bath (Figure 1), the colon was incubated with Fluo-4-AM (Molecular Probes; Eugene, Oregan; U.S.A) for 20 min at room temperature, whilst constant bubbling with oxygenated Krebs solution (see composition below). Following the loading procedure, the preparation was perfused with warmed Krebs solution at 36°C, to allow for de-esterification. The imaging set-up consisted of a Nikon eclipse 50i upright microscope, fitted with epi-fluorescence FITC filter cubes with an excitation and emission wavelength of 492 and 520 nm, respectively. Fluo-4 AM has a peak excitation at 485 nm and peak emission at 525 nm. For all imaging experiments of nerve endings, water immersion (10 × 0.3 W, 20 × 0.5 W & 40 × Fluor; 0.8 W) lens were used, where the working distance of each lens varied from 3.5 to 2.0 mm, respectively). An electron multiplied (EM-CCD) camera (Cascade II 512, Roper Scientific, Tucson, AZ, U.S.A) was used to record dynamic changes in fluorescence activity from spinal nerve terminals. We acquired data at 40–50 Hz, (image capture interval of 20–25 ms). All imaging data was acquired and analyzed using Imaging Workbench (Version 6.0; INDEC BIOSYSTEMS; Santa Clara; California).

In all experiments, the colon was perfused with hexamethonium (400 μM), which abolishes all spontaneous fast synaptic potentials in myenteric neurons (Furukawa et al., 1986; Nurgali et al., 2004); and in the presence of nifedipine (1 μM) to abolish calcium action potentials, through L-type voltage dependent calcium channels in the smooth muscle.

### Analysis of calcium transients in spinal nerve terminals

The analysis of calcium transients in spinal nerve terminals was also made using Imaging Workbench software (Version 6.0; INDEC BIOSYSTEMS; Santa Clara; California). The rise time and half duration of each calcium transient was measured, as was the intervals between calcium transients. The use of “*N*” in the results section refers to the number of animals on which observations were made. Ca^2+^-induced fluorescence changes we describe represent the average intensity with a region of interest. When stretch-induced firing of spinal afferent endings was identified in a field of view, we identified multiple regions of interest around discrete varicosities and the axons between varicosities. This enabled us to record and compare the dynamic changes in fluorescence in both structures. The time-to-peak and half duration of calcium transients were measured using Workbench, version 6.0. The single calcium indicator, Fluo-4 was used for all experiments, which was excited at one wavelength (~490 nm) and the emission observed at ~520 nm. The relative changes in fluorescence were reported as delta *F*/*F*_*o*_, where delta F stands for the change in fluorescence intensity and *F*_*o*_ for the baseline level. Because only one wavelength of light was used, calibrating the absolute concentration of calcium was practically impossible, consistent with other studies. Nevertheless, the single wavelength of light used was sufficient for relative changes, or non-quantitative changes in fluorescence. Hence, our fluorescence changes are reported as delta *F*/*F*_*o*_.

### Protocol for testing effects of circumferential stretch on rectal afferent nerve endings

Investigations were made to determine whether application of circumferential stretch to the colon would increase the excitability of spinal afferent nerve endings, such that the activity of these nerve endings could be visualized and recorded for the first time in the gut wall. To test this, we recorded from full length isolated colons that were pinned slack with no imposed circumferential stretch, to the base of the Sylgard-lined organ bath. We then imposed a 40% increase in the circumferential slack (resting) diameter of the entire colon and pinned the entire full length of colon to the base of the organ bath. Calcium imaging was then made from these stretched preparations.

### Immunohistochemical staining for TRPV1 in mouse colorectum

#### Fixation and dissection

Whole mouse colon preparations were fresh fixed by immersing overnight in Zamboni fixative at 4°C. Prior to fixation, the colon was pinned out under tension, in a Sylgard lined Petri dish (Dow Corning Corp). After clearing in dimethyl sulfoxide (10 min immersion, repeated three times), the colon was washed in phosphate buffered saline [phosphate buffered saline (PBS); 0.2 mol L^−1^ sodium phosphate buffer, pH 7.2] (10 min immersion, repeated three times). A whole mount of the circular muscle, myenteric plexus and longitudinal muscle was prepared by removing the mucosa and submucosa in a solution of phosphate buffered saline with 1% Triton X-100 and the aid of a dissecting microscope.

#### Immunohistochemistry

Specimens were labeled by incubation with antisera to TRPV1 (VR1) sourced from Alomone Labs, Product #: ACC-030 for 2 days at a concentration of 1:500, then washed (10 min immersion, repeated three times) in PBS. Secondary antisera were then applied (donkey anti rabbit CY5 Jackson Immunoresearch Laboratories Inc.) for a further 2 h at a concentration of 1:200 at room temperature then washed (10 min immersion, repeated three times) in PBS. Specimens were mounted in 100% Buffered Glycerol.

### Drugs and solutions

The composition of the KRB was in (mM): NaCl, 118; KCl, 5.9; NaHCO_3_, 15.5; NaH_2_PO_4_, 1.2; MgSO_4_, 1.2; CaCl_2_, 2.5; and glucose, 11.5. Tetrodotoxin (TTX), Cyclopiazonic acid (CPA), BAPTA-AM, Hexamethonium Bromide, Gadolinium, NMDG and Nicardipine were all obtained from Sigma Chemical Co. (St. Louis, MO, USA). To test for the involvement of non-selective, stretch-activated cationic channels in the generation of calcium transients, we replaced NaCl with an equal concentration (118 mN) of N-methyl-D-glucamine (NMDG) solution. Addition of NMDG led to a significant increase in pH in the range of 8.5–9.0. To correct for this, we restored the pH to 7.4 with small quantities of 10 M HCl solution. For Ca^2+^ imaging, a solution consisting of 25 μg Fluo-4 (FluoroPureTM-AM, Molecular Probes, Eugene, OR), 0.02% DMSO and 0.01% non-toxic detergent Cremophor EL was used.

## Results

### Effects of circumferential stretch on nerve endings of rectal afferents in myenteric ganglia and circular muscle

**Figure 2:**
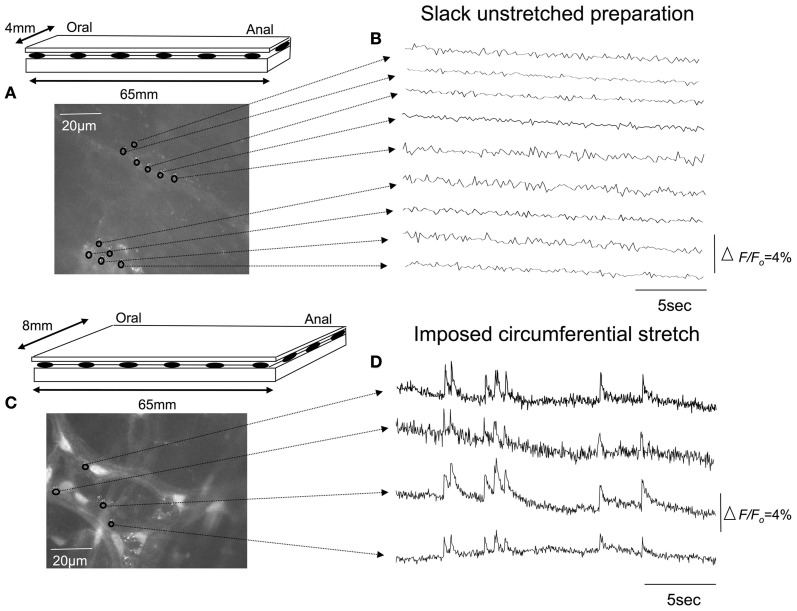
**Effects of circumferential stretch on rectal afferent nerve endings. (A)** Diagrammatic representation of the isolated full length colon. The preparation was pinned unstretched, to a resting circumferential diameter of 4 mm and length (slack) of 65 mm. **(B)** Shows calcium recordings from inactive regions of interest throughout myenteric ganglia. **(C)** In the same segment of colon, increasing the circumferential diameter to 8 mm, without changing the longitudinal length of the preparation led to a discharge of calcium transients in Panel **(D)**. The relative changes in fluorescence were reported as delta *F*/*F*_*o*_, where delta *F* stands for the change in fluorescence intensity and *F*_*o*_ for the baseline level.

**Figure 3:**
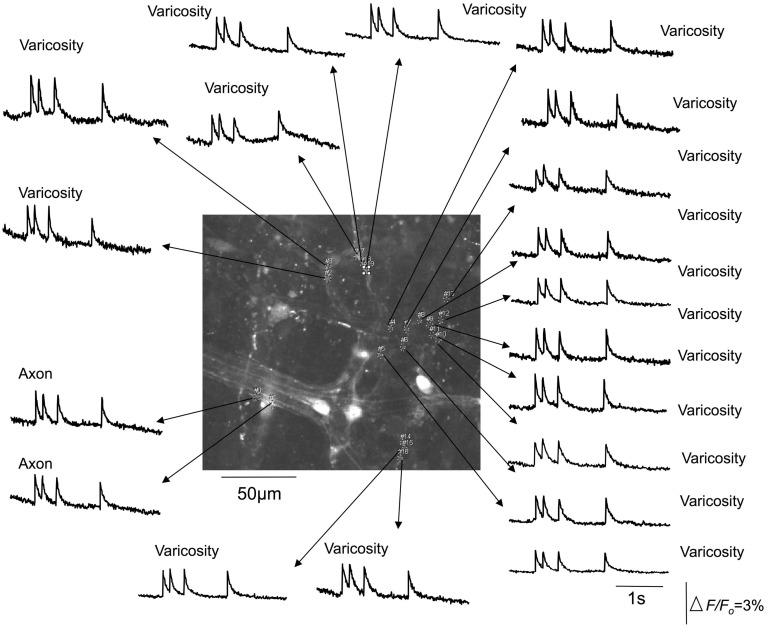
**Calcium transients in spinal afferent nerve terminals that ramify within a myenteric ganglia of a stretched segment of isolate mouse colon.** Calcium transients discharge simultaneously in multiple varicosities that exist along a single axon. Note, the axon also generates a calcium transient at the same time as the activation of the varicosities. The relative changes in fluorescence were reported as delta *F*/*F*_*o*_, where delta *F* stands for the change in fluorescence intensity and *F*_*o*_ for the baseline level.

We used the calcium indicator, Fluo-4 to image the nerve endings of spinal afferents that have been shown previously to innervate myenteric ganglia and circular muscle of isolated whole mouse colon. To test this, we made 10 independent recordings from 7 full length preparations of colon that were pinned unstretched (slack) to the base of the Sylgard-lined organ bath, with a mean circumferential diameter of 4.3 ± 0.2 mm (*N* = 7). In all unstretched segments of colon, no calcium transients were ever detected in any myenteric ganglia or circular smooth muscle (70 recordings; *N* = 7 animals). From the same colonic preparations, we then increased the mean circumferential diameter to 8.1 ± 0.2 mm, and pinned the preparations fixed to the base of the organ bath, to determine the effects of maintained circumferential stretch. In these stretched preparations, an ongoing discharge of calcium transients was detected in multiple varicosities simultaneously along single spinal afferent axons that ramified extensively throughout myenteric ganglia (Figures 2, 3; See Movie S1); and in intramuscular endings within circular smooth muscle (see Movie S2). All calcium transients were resistant to hexamethonium (500 μM) applied to the colon, which has been shown to completely block spontaneous enteric synaptic transmission (Furukawa et al., 1986; Nurgali et al., 2004).

It was noted that the temporal activation of calcium transients in discrete varicosities was synchronized in time with calcium transients that occurred in the axon between varicosities (see Figures 2, 3). The mean firing interval between calcium transients was 2.8 ± 0.05 s; while the shortest interval (maximum frequency) between calcium transients was 1.4 ± 0.04 s (*N* = 14). Tables 1 and 2 shows the mean half duration and time-to-peak of individual calcium transients in myenteric and intramuscular terminals recorded from axons and varicosities. Examples of synchronized calcium transients in a single axon and varicosities from a single axon of a rectal afferent terminal that innervates a myenteric ganglion is shown in Movie S1; and an intramuscular ending in Movie S2.

**Table 1:** **Characteristics of half duration of calcium transients in varicosities and axons of spinal nerve terminals that ramify within myenteric ganglia and circular muscle**.

	**Myenteric nerve terminals**		**Intramuscular nerve terminals**	
	**Varicosities**	**Axons**	**Varicosities**	**Axons**
Mean half duration (ms)	137.8	111.6	108.4	92.6
SEM	4.2	6.4	2.9	2.6
Number of observations	236 events (*N* = 14)	88 events (*N* = 14)	129 events (*N* = 14)	126 events (*N* = 14)

**Table 2:** **Characteristics of time-to-peak of calcium transients in varicosities and axons of spinal afferent nerve terminals that ramify within myenteric ganglia and circular muscle**.

	**Myenteric nerve terminals**		**Intramuscular nerve terminals**	
	**Varicosities**	**Axons**	**Varicosities**	**Axons**
Mean time to Peak (ms)	82.6	92.3	72.1	74.1
SEM	3.1	11.2	2.7	3.5
Number of observations	236 events (*N* = 14)	88 events (*N* = 14)	129 events (*N* = 14)	78 events (*N* = 14)

The mean half duration of calcium transients in myenteric varicosities was 137.8 ± 0.3 ms and in myenteric axons was 111.6 ± 0.7 ms (88 events, *N* = 14), while the mean half duration of calcium transients in intramuscular CM varicosities was 101.3 ± 0.4 ms and intramuscular axons was 88.7 ± 1.1 ms (88 events, *N* = 14). We spritzed capsaicin (5 μM) directly onto stretch-activated nerve endings in an attempt to pharmacologically activate these endings. This turned out to yield non-specific activation of many different cell types, probably because of release of CGRP from spinal nerve endings. Also, capsaicin directly activated blood vessels, which also are now known to express TRPV1 receptors (Cavanaugh et al., 2011).

### Effects of tetrodotoxin on calcium transients in spinal afferent nerve terminals

**Figure 4:**
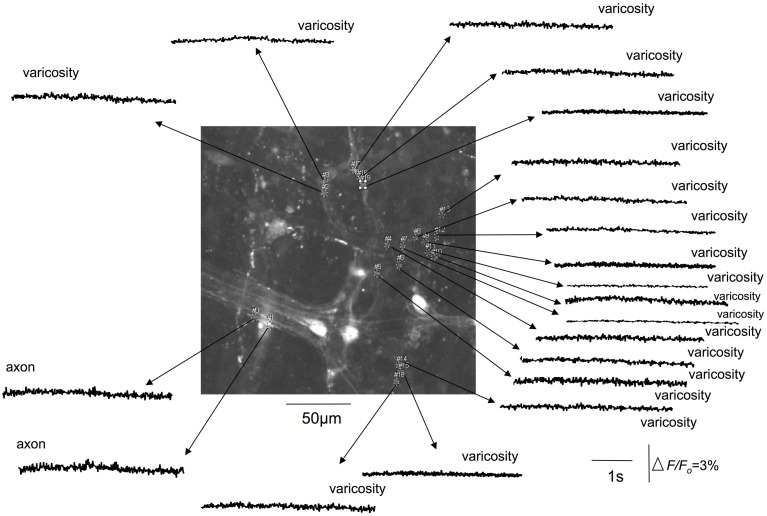
**Tetrodotoxin abolishes stretch-activated calcium transients in varicosities and axons of spinal afferent nerve terminals that innervate the same myenteric ganglion, as shown in Figure 3.** The relative changes in fluorescence were reported as delta *F*/*F*_*o*_, where delta *F* stands for the change in fluorescence intensity and *F*_*o*_ for the baseline level.

We investigated whether TTX would modify the stretch-activated discharge of calcium transients in myenteric ganglia and intramuscular nerve endings in circular muscle, since TTX-resistant sodium channels can play a major role in generating action potentials in nociceptive nerve terminals (Brock et al., 1998, 2001; Carr and Brock, 2002; Carr et al., 2009). It was found that TTX at 1 μM consistently abolished all axonal and varicose calcium transients within myenteric ganglia (*N* = 5; Figure 4) and intramuscular nerve endings that ramified within circular muscle. We confirmed that the abolition of calcium activity by TTX was due to the TTX itself; and not due to any natural steady decay in the fluorescence signal in nerve endings. To test this, we measured the percentage reduction in intensity of calcium transients over a continuous 5 min illumination period. It was found that the amplitude of the mean delta *F*/*F*_*o*_ over 5 continuous minutes of illumination was reduced by 33% from the control value. In contrast, when TTX entered the organ bath, it completely abolished all calcium transients in varicosities and axons of spinal afferent nerve endings within 40–55 s after entering the recording bath (*N* = 5). To eliminate any possibility that natural decay in fluorescence interfered with our interpretation of the effects of any antagonists applied, we typically recorded stretch-activated calcium transients for no longer than 2 min before any antagonists were exposed to colonic preparations. We could not illuminate preparations for more than 15 min continuously, without bleaching calcium signals in our preparations, consistent with our previous studies. Hence, since washout of the organ bath and the preparation took many minutes, testing recovery of stretch-induced calcium transients after washout of antagonists was unfortunately not a realistic option.

### TRPV1 immunoreactivity in mouse colon

**Figure 5:**
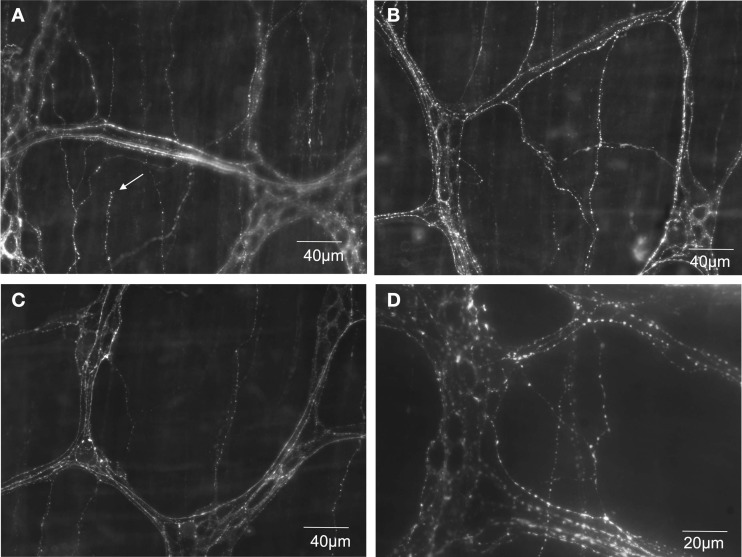
**TRPV1 immunoreactive nerve endings identified in mouse colonic myenteric ganglia and circular smooth muscle. (A–C)** Show dense TRPV1 varicose fibers innervate myenteric ganglia and circular muscle. The arrow in **(A)** shows the ending of a varicose TRPV1 positive nerve ending that lacks complex morphological specialization. **(D)** Shows a higher magnification (40× water immersion lens) of the dense TRPV1 positive varicose axons that ramify within myenteric ganglia.

The capsaicin receptor, TRPV1 is an established marker for spinal afferents in the gastrointestinal tract (Robinson et al., 2004). We used an antibody to the TRPV1 receptor to verify the location of TRPV1 positive nerve terminals in the mouse colon. It was found that extensive TRPV1 positive varicose nerve terminals were identified in the circular muscle layer (see arrow in Figure 5A) and myenteric ganglia (Figure 5D). In the circular muscle, the TRPV1 immunoreactive fibers could be identified as simple nerve endings, with bulbous endings (Figure 5), that were highly reminiscent of “simple type” nociceptive afferent endings described recently in the cornea; see (Ivanusic et al., 2012).

### Confirmation that stretch-activated calcium transients in varicose axons in myenteric ganglia and circular muscle were of spinal afferent origin

**Figure 6:**
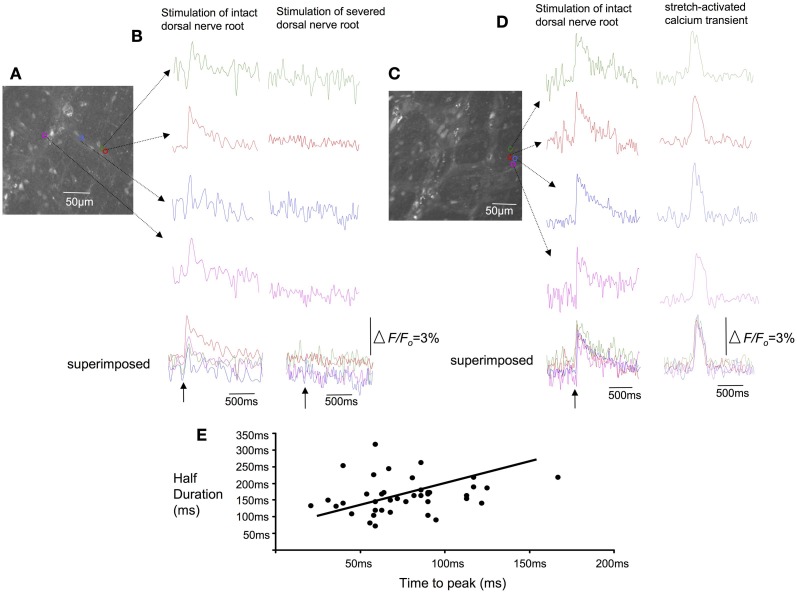
**Effects of selective electrical nerve stimulation applied to dorsal (afferent) nerve roots on calcium induced fluorescence changes in rectal afferent nerve endings in colonic myenteric ganglia. (A)** Photomicrograph of the region of interest from which calcium transients were recorded in this single myenteric ganglia. **(B)** Single pulse (70 V, 0.4 ms) electrical stimuli applied to dorsal nerve roots arising from L6-S2 evoked brief calcium transients in axon varicosities within myenteric ganglia (left hand panel). When the dorsal roots were lesioned (right hand panel), the same stimulus failed to evoke any response. **(C)** Shows a micrograph of a single myenteric ganglion from another preparation. **(D)** Electrical stimulation (single pulse: 0.4 ms, 70 V) applied to L6-S1 DRG again evoked a calcium transient in individual varicosities from a single myenteric ganglia (left hand side of figure). In the same regions of interest, a stretch-activated calcium transient (see right hand side of the recording) occurs with similar time course to the electrically-evoked transient. **(E)** Shows the characteristics of calcium transients evoked in varicose fibers of myenteric ganglia in response to single pulse electrical stimulation of dorsal nerve roots. As the time-to-peak of individual calcium transients increases, there is a trend that the half duration of each transient also increases. The relative changes in fluorescence were reported as delta *F*/*F*_*o*_, where delta *F* stands for the change in fluorescence intensity and *F*_*o*_ for the baseline level.

**Figure 7:**
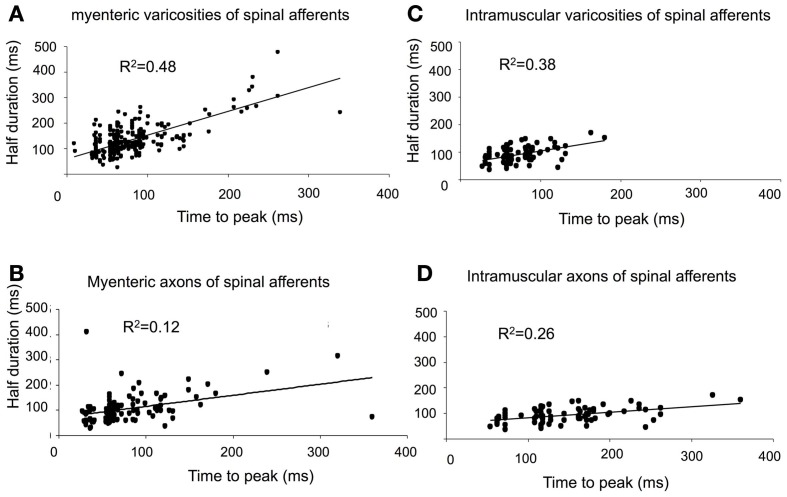
**Characteristics of calcium transients in varicosities and axons of spinal afferents. (A,B)** Shows temporal characteristics of time-to-peak of calcium transients in myenteric varicosities **(A)** and axons **(B)** plotted against the half duration of individual calcium transients. Panels **(C)** and **(D)**, show the same characteristics plotted for intramuscular nerve terminals in the circular muscle for **(C)** varicosities and **(D)** axons. As the time-to-peak of individual calcium transients increases, so to do the half duration of each transient.

In all our experiments, hexamethonium (500 μM) was present in the colon which blocks ongoing synaptic potentials and action potentials in myenteric ganglia (Furukawa et al., 1986; Nurgali et al., 2004). However, to confirm that the stretch-activated calcium transients in varicose axons in myenteric ganglia and circular muscle arose from spinal afferent neurons, whose cell bodies arose from DRG, we selectively stimulated dorsal nerve roots with transmural electrical nerve stimuli (see Figure 1). These nerve roots consist purely of spinal afferent axons. We found that electrical stimulation of the dorsal nerve roots that emanated from the right bank only of L6-S1 DRGs, elicited a calcium transient in 38% of the same single varicose axons that demonstrated stretch-evoked calcium transients. Because only the right bank of DRGs from L6-S2 were preserved with the colon, this meant that we could not have ever evoked a calcium response in more than ~50% of stretch-activated nerve endings arising from these DRGs. Stimulation of these dorsal nerve roots with a single pulse (80 V, 0.4 ms duration) evoked a calcium transient whose time course closely mimicked that of stretch-activated calcium transients in single varicose spinal axons (c.f. Figures 3, 6, 7). The mean half duration of calcium transients evoked by single pulse electrical nerve stimulation was 88.9 ± 13.3 ms, where the time-to-peak of these events was 161 ± 8.0 ms (*N* = 5; Figure 6). Since the lumbar colonic and hypogastric nerves were severed prior to stimulation of dorsal roots (see Figure 1), the activation of the calcium transients in afferent endings in the colon must have been transmitted from DRGs via pelvic/rectal afferent pathway to the colon. When the time to peak of discrete electrically-evoked calcium transients was plotted against their half duration, it was found that there was a similar correlation to stretch-activated calcium transients in rectal afferent nerve endings such that as the time-to-peak increased, there was an increase in their half duration.

### Effects of non-selective stretch activated ion channel blockers on stretch-evoked firing of rectal afferent nerve endings

Since experiments above showed that circumferential stretch was required for the synchronized firing of calcium transients in rectal afferent nerve endings, we were particularly interested in whether blockers of stretch-activated ion channels might affect this activity. Initially, we tested the effects of gadolinium (50 μM). In stretched preparations, it was found that gadolinium blocked stretch-activated firing in rectal nerve endings that innervated myenteric ganglia and circular muscle (*N* = 5). To further investigate the effects of non-selective cationic channels in stretch-induced firing of rectal afferents, we investigated the effects of replacing extracellular Na^+^ with an equal concentration of NMDG (118 mN). In a separate cohort of experiments (*N* = 5), it was found that stretch-activated firing of calcium transients in rectal afferents were also abolished by NMDG (118 mM). In one of 5 animals, calcium transients returned upon washout of NMDG.

### Effects of cobalt, cyclopiazonic acid (CPA) and BAPTA-AM on synchronized calcium transients in spinal afferent terminals

**Figure 8:**
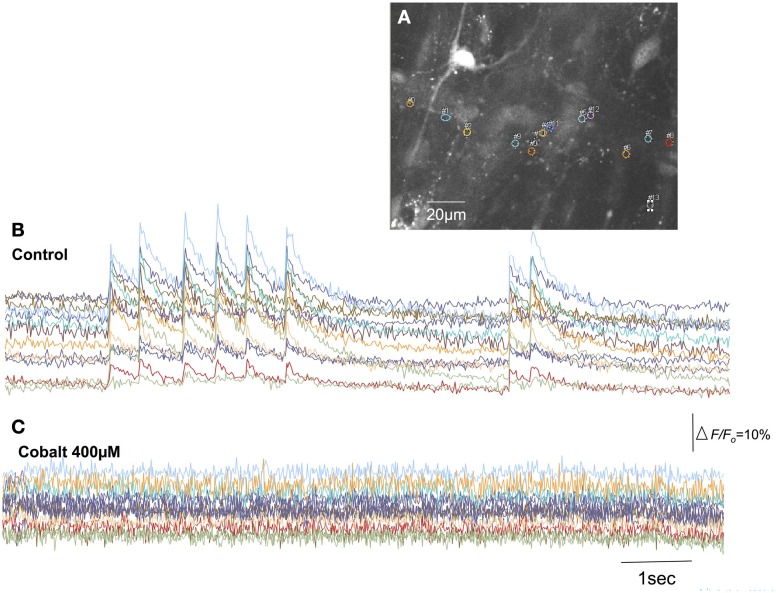
**Blockade of extracellular calcium influx through voltage-sensitive Ca^2+^ channels with cobalt abolishes calcium transients in spinal afferent nerve terminals. (A)** Shows the camera image of a single myenteric ganglion in which calcium transients were detected in single varicosities along a single spinal axon. **(B)** Shows calcium transients in varicosities represented by the colored regions of interest. **(C)** The discharge of calcium transients was abolished by cobalt (400 μM). The relative changes in fluorescence were reported as delta *F*/*F*_*o*_, where delta F stands for the change in fluorescence intensity and *F*_*o*_ for the baseline level.

In this series of experiments, we sought to investigate whether intracellular or extracellular calcium stores contributed to the activation of spinal afferent nerve terminals that innervated the myenteric ganglia and circular muscle layer. To determine whether extracellular calcium influx was required for the calcium transients detected in spinal afferent terminals we applied cobalt 300 μM to the colon chamber. After 20–30 min perfusion, no calcium transients were ever detected in myenteric ganglia (*N* = 5; Figure 8) or circular smooth muscle (*N* = 5). We then tested whether release of calcium from intracellular stores might contribute to the generation of calcium transients in sensory nerve endings that ramified within circular muscle and myenteric ganglia. It was found that CPA 5 μM, to block calcium uptake from the sarcoplasmic reticulum abolished all calcium transients both intramuscular (*N* = 5) and myenteric origin (*N* = 5). Also, in a separate cohort of animals (*N* = 5), buffering intracellular Ca^2+^ with BAPTA-AM (30 μM) had a similar blocking effect on both intramuscular (*N* = 5) and myenteric class of nerve terminals (*N* = 5).

## Discussion

In this study, we developed an isolated DRG-colon preparation, in which calcium imaging can be used to record the activation of spinal afferent nerve endings, in response to circumferential stretch of the mouse colon. Spinal afferent nerve endings arising from the rectal/pelvic pathway are of supreme interest to us, because these afferents have been shown to underlie the detection and transmission of visceral pain from the colorectum, see (Kyloh et al., 2011). A major finding of the current study is that in response to maintained circumferential stretch of the colon, brief calcium transients (with a mean unitary half duration <140 ms) can be recorded in multiple varicosities simultaneously along single rectal afferent nerve endings. These nerve endings ramified extensively within myenteric ganglia and circular muscle. These calcium transients discharged continuously whilst the colon was maintained under circumferential stretch. Similar firing was not recorded from the same preparations, when no circumferential stretch was imposed on the colon.

### Characteristics of calcium transients in spinal afferent nerve endings and their relationship to neuronal action potentials

A major finding of the current study was that opening of stretch-activated, non-selective cation channels is required for the synchronized discharge of calcium transients in multiple varicosities of rectal afferent endings. Our findings also reveal that, intracellular and extracellular calcium plays a vital role in generating stretch-activated calcium transients in spinal afferent endings. This was not known prior to these recordings. These observations lead us to suggest that the simultaneous discharge of calcium transients in multiple varicosities of rectal afferent endings require the presence of TTX-sensitive action potentials, elicited by circumferential stretch of the large intestine.

Tetrodotoxin was found to abolish calcium transients in varicosities and axons of rectal afferent nerve endings that ramified within myenteric ganglia and circular muscle layer. This showed that TTX-sensitive Na^+^ sodium channels are required for activation of calcium transients. It does not exclude the possibility that TTX-resistant sodium channels are also involved in the generation of the nerve terminal action potentials or calcium transients. A role for TTX-resistant Na^+^ channels is entirely plausible in the stretch-evoked firing of rectal afferent endings. However, in our hands, these channels alone are insufficient to activate the detectable calcium transients in varicosities and axon terminals, since blockade of TTX-sensitive channels alone abolished all *detectable* calcium transients.

### Characteristics of stretch-activated firing of spinal afferent nerve endings and the nature of our recording conditions

All our experiments were performed in the presence of nifedipine and hexamethonium, applied to the large intestine, to paralyze smooth muscle contractions and block enteric synaptic transmission. The fact that calcium transients discharged in the presence of nifedipine confirms that L-type Ca^2+^ channels are not required for the activation of the nerve endings of spinal afferents in response to circumferential stretch, nor is contraction of the smooth muscle. In our imaging system, we acquired data with a frame rate of 40–50 frames per second. This meant that the shortest interval between acquiring fluorescent images was separated by 20 ms. However, the duration of enteric (Michel et al., 2011) and spinal neuronal action potentials (Spencer et al., 2008a; Song et al., 2009; Michel et al., 2011; Feng et al., 2012) is typically <5 ms. This means that the calcium transients we recorded with our acquisition system are unlikely to be due to calcium that enters during a *single* action potential. It is possible, however, that our recording technique could capture calcium influx entering nerve terminals from the extracellular space in response to multiple action potentials. Indeed, our observation that cobalt abolished all calcium transients is highly indicative that extracellular calcium influx across the plasma membrane contributes to the calcium transients recorded from rectal nerve terminals. Interestingly, when intracellular calcium stores were depleted by CPA, stretch-activated calcium transients were also abolished. This shows that without intracellular calcium stores, calcium transients also cannot be detected, even if extracellular influx of calcium entry is preserved. The most likely explanation for our findings is that calcium crosses the plasma membrane during action potentials and that this calcium leads to release of calcium from intracellular stores. However, blockade of either intracellular calcium stores, or, extracellular influx alone did not allow any residual calcium to be detected in these nerve endings. At present, we obtained no data to suggest that we can record the generator potential in rectal afferent terminals. The calcium transients recorded in this study are likely elicited as a consequence of neuronal action potentials in these terminals.

### Technique to confirm calcium transients arose from spinal afferent terminals

One of the major challenges with recording from spinal afferent nerve terminals in the gastrointestinal tract is the difficulty in identifying which axons belong specifically to spinal afferents, and which axons belong to enteric neurons, or, other extrinsic afferent or efferent fibers. To confirm that the calcium transients in varicose axons in the colon arose from spinal afferents in the rectal nerves, we selectively stimulated the dorsal nerve roots that emanate from the dorsal horn of the spinal cord (Figure 1). The dorsal nerve roots consist only of spinal afferent axons. Therefore, when a calcium transient is detected in the colon, as a result of dorsal root electrical nerve stimulation, the varicose nerve ending that is activated in the colon must be a spinal afferent. Our preparations consisted only of the right bank of DRGs (from L6-S2) with the colon. This meant that we could not have ever evoked a calcium response in more than 50% of stretch-activated nerve endings arising from these DRGs. Overall, we found that 38% of preparations in which stretch-activated calcium transient discharged in afferent endings, these same endings responded to dorsal nerve root stimulation (Figure 6). Why a higher proportion of axons and varicosities, that were activated by stretch, did not also respond to dorsal nerve root stimulation could be due to: (1) the nerve ending that we were imaging did not have its endings in the field of view at that point in time. Or, alternatively, the nerve ending that we were imaging may have had its cell body in a DRG that was not within the L6-S2 range that we extracted from the colon. Nevertheless, our data shows that a substantial proportion of the nerve endings that were activated by circumferential stretch, must be spinal afferents. This conclusion was further confirmed by lessoning the rectal nerves, which abolished all calcium transients in the colon, following electrical stimulation of dorsal nerve roots. Importantly, we had lesioned the hypogastric and lumbar colonic nerves in all our experiments (Figure 1). This meant that any calcium transients evoked in the colon, as a consequence of DRG or dorsal nerve root stimulation could only have been due to activation of rectal afferents, via the pelvic nerve pathway (Figure 1). This rectal/pelvic afferent pathway has been shown *in vivo* to directly underlie nociception, see (Kyloh et al., 2011).

### How do the current findings relate to what is known electrophysiologically about the activation of spinal afferent nerve terminals innervating the large intestine?

**Figure 9:**
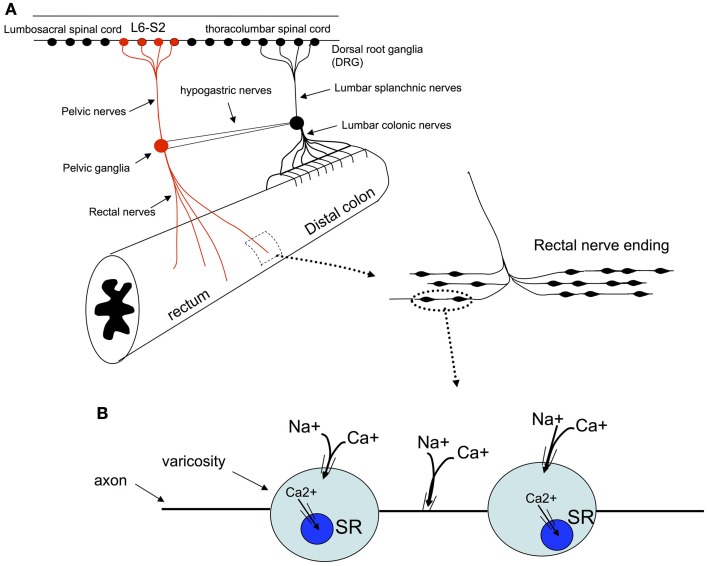
**Schematic representation of the proposed mechanisms by which colonic circumferential stretch activates synchronized calcium transients in rectal afferent nerve terminals. (A)** Shows the primary pain pathway from the mouse colorectum, see (Kyloh et al., 2011). **(B)** It is suggested that circumferential stretch of these nerve endings leads to the opening of non-selective cationic channels which lead to the generation of action potentials. Activation of action potentials in these nerve endings is the likely stimulus for the synchronous calcium transients in multiple varicosities and axons of rectal afferent endings. TTX-sodium channels are required for activation of calcium transients, as are intracellular calcium stores and extracellular calcium influx.

In previous studies, it has been shown that depletion of intracellular and extracellular calcium does not prevent activation of spinal afferent nerve endings in the colon (Lynn et al., 2005). This meant that calcium *per se* was not required for the mechanotransduction process that initiates action potentials in spinal afferent nerve endings in the colon. However, it is clear that total depletion of intracellular and extracellular calcium changes substantially the excitability of spinal afferents to mechanical stimuli (Spencer et al., 2008a,b), suggesting that calcium handling mechanisms play an important role in controlling the excitability of the mechanotransduction process. The findings of the current study show that blockade of extracellular calcium influx with cobalt, or blockade of intracellular calcium stores with CPA or BAPTA-AM, abolishes the discharge of calcium transients in rectal afferent nerve terminals. This suggests that blockade of extracellular calcium influx across the plasma membrane, or blockade of intracellular calcium stores is essential for the activation of calcium transients in varicosities, and axons between varicosities, of single fiber afferent terminals (Figure 9). Whilst no similar studies have been performed on visceral afferents, previous studies on mouse postganglionic (efferent) sympathetic axon bundle have recorded calcium transients using confocal imaging (Jackson et al., 2001). These studies revealed that calcium transients in axons were found to be dependent upon voltage dependent Na^+^ channels but interestingly were unaffected by depletion of intracellular calcium stores (Jackson et al., 2001). It was concluded that calcium transients originated primarily via calcium entry through voltage sensitive calcium channels. These findings differ considerably from our results in primary afferent nerve terminals, where blockade of intracellular calcium stores abolished all calcium transients in both varicosities and axons of rectal afferents.

## Conclusions

We demonstrate that calcium imaging can be used to record dynamic changes in intracellular calcium from multiple sites simultaneously along rectal afferent nerve terminals that respond to maintained stretch. Until now, all previous recordings from spinal afferents have been from axons or cell bodies, that lie outside the visceral organ, which is a considerable distance away from the site where mechanotransduction and action potential initiation occurs.

We show that in response to maintained circumferential stretch, opening of non-selective stretch-activated cation channels is required for the synchronized discharge of calcium transients in multiple varicosities along single rectal afferent endings that innervate myenteric ganglia and circular muscle. Stretch-activated calcium transients in varicosities and axons of this class of rectal afferent require TTX-sensitive Na^+^ channels, and both intracellular calcium stores and extracellular calcium influx (Figure 9). This novel preparation will facilitate deeper investigations into the mechanisms underlying activation of spinal afferent varicosities in response to circumferential stretch. An exciting challenge will be to characterize the specific identity of the stretch-activated ion channels in this, and other classes of spinal afferent, that innervate the colon.

## Funding and disclosures

This work was supported by a grant # 1025766 from the National Health & Medical Research Council (NH&MRC) of Australia to Nick J. Spencer.

## Conflict of interest statement

The authors declare that the research was conducted in the absence of any commercial or financial relationships that could be construed as a potential conflict of interest.

## Supplementary material

Movie S1 **This movie shows a discharge of calcium transients that occurs in two independent spinal afferent axons that both ramify within a single myenteric ganglion of the distal colon, that was pinned under maintained circumferential stretch**. A 40× water immersion lens was used to capture the images at a frame rate of 40 Hz; and the movie is reproduced in real time. The first axon shows a discharge of calcium transients that occurs simultaneously in multiple discrete varicosities along a single axons (indicated by the arrows appearing toward the start of the movie), whilst in the presence of hexamethonium (400 μM) and nifedipine (1 μM). The first axon that is activated is fires throughout the movie. Toward the end of the movie, a second independent axon and associated varicosities are also activated simultaneously (indicated by different arrows appearing toward the end of the movie).

Movie S2 **This movie shows a discharge of calcium transients in a single intramuscular spinal afferent axon that innervates circular muscle, from a preparation of mouse colon pinned under maintained circumferential stretch**. The data was acquired at using a frame rate 40 Hz and the movie reproduced in real time. The discharge of calcium transients occurs in discrete varicosities that are temporally synchronized with calcium discharges in the axon from which the varicosities arise. The recording was made in the presence of hexamethonium (400 μM) and nifedipine (1 μM) applied to the colon. The arrows that appear on the screen indicate the location of varicosities.
